# National healthcare-associated infections surveillance programs: A scoping review

**DOI:** 10.14745/ccdr.v48i78a05

**Published:** 2022-07-07

**Authors:** Etienne Poirier, Virginie Boulanger, Anne MacLaurin, Caroline Quach

**Affiliations:** 1 Department of Microbiology, Infectious Diseases, and Immunology, Faculty of Medicine, University of Montréal, Montréal, QC; 2 Research Center, CHU Sainte-Justine, Montréal, QC; 3 Healthcare Excellence Canada; 4 Infection Prevention & Control, CHU Sainte-Justine, Montréal, QC; 5 Department of Pediatric Laboratory Medicine, CHU Sainte-Justine, Montréal, QC

**Keywords:** healthcare-associated infections, surveillance, surgical site infections, *Clostridoides difficile*, Methicillin-resistant *Staphylococcus aureus*

## Abstract

**Background:**

National surveillance of healthcare-associated infections (HAIs) is necessary to identify areas of concern, monitor trends, and provide benchmark rates enabling comparison between hospitals. Benchmark rates require representative and large sample sizes often based on pooling of surveillance data. We performed a scoping review to understand the organization of national HAI surveillance programs globally.

**Methods:**

The search strategy included a literature review, Google search and personal communications with HAI surveillance program managers. Thirty-five countries were targeted from four regions (North America, Europe, United Kingdom and Oceania). The following information was retrieved: name of surveillance program, survey types (prevalence or incidence), frequency of reports, mode of participation (mandatory or voluntary), and infections under surveillance.

**Results:**

Two hundred and twenty articles of 6,688 identified were selected. The four countries with most publications were the US (48.2%), Germany (14.1%), Spain (6.8%) and Italy (5.9%). These articles identified HAI surveillance programs in 28 of 35 countries (80.0%), operating on a voluntary basis and monitoring HAI incidence rates. Most HAIs monitored surgical site infections in hip (n=20, 71.4%) and knee (n=19, 67.9%) and *Clostridoides difficile* infections (n=17, 60.7%).

**Conclusion:**

Most countries analyzed have HAI surveillance programs, with characteristics varying by country. Patient-level data reporting with numerators and denominators is available for almost every surveillance program, allowing for reporting of incidence rates and more refined benchmarks, specific to a given healthcare category thus offering data that can be used to measure, monitor, and improve the incidence of HAIs.

## Introduction

Healthcare-associated infections (HAI) are acquired by patients during the process of care for other health conditions ((1)). They are the most frequently reported adverse event in healthcare delivery ((2)), affecting millions of patients each year worldwide and leading to significant morbidity, mortality and financial costs to healthcare programs. In the beginning of 2000, HAI prevalence in high-income countries ranged between 3.5% and 12%; in Europe, for example, the average prevalence is 7.1%, representing over four million people infected each year ((3)).

The emergence of antimicrobial-resistant organisms (AROs) complicates the situation, making HAIs more difficult to treat. The Public Health Agency of Canada estimated that approximately 2% of patients admitted to large academic Canadian hospitals will have acquired an infection during their hospital stay ((4)) and that at any time, 3%–10% of hospitalized patients are either infected with or a carrier of an ARO ((5)).

Surveillance of HAIs is considered a necessary component of infection prevention and control, public health and patient safety. National surveillance requires representative and large enough sample sizes to produce meaningful infection rates for benchmarking, detection of trends and prioritization of interventions at a regional or local level, and for specific populations.

Many countries have national HAI surveillance programs, but a comprehensive review of these countries’ program characteristics is not currently available. We conducted a scoping review to identify national HAI surveillance programs globally and summarized their characteristics to inform decisions on possible national programs for Canada.

## Methods

### Research question

The main research question was: What are the characteristics of HAI surveillance programs in a selected sample of high-income countries, defined by the World Bank as countries with a gross national income per capita of at least US$12,696 ((6)) We added the following sub-questions to have a more complete picture: Is the program mandatory or voluntary? Is it based on incidence or prevalence analysis? What are the infections or procedures under surveillance? What is the frequency of public reporting?

### Scoping review

The first step was a scoping review using Medline. We performed a search strategy developed with a medical research librarian. Keywords and MeSH were created in Medline with the following four concepts: nosocomial, epidemiology, surveillance and administration (Table S1). The inclusion criteria consisted of articles identifying surveillance of HAIs in four selected high-income regions in the world: North America, Europe through the European Centre for Disease Prevention and Control (ECDC), United Kingdom (UK) and Oceania. The ECDC encompassed 27 countries (26 countries and ECDC itself), for a total of 35 countries in these four regions. Surveillance needed to be reported at the national level. We included articles published between January 1, 1996, and December 31, 2020, written in English or French. Government publications or reports and grey literature that contained any surveillance data on HAI were kept. Opinion, editorial, news reports, abstracts from conferences or meetings were excluded. Only human health articles were considered. We searched Medline, grey literature and communicated with key people.

### Grey literature

Grey literature was used to compile unidentified HAI surveillance programs from Medline. National organizations’ websites of the four regions cited in the inclusion criteria were considered. Once the program name was retrieved, usually from published articles, a Google search was performed to get publicly available information on the HAI surveillance program, aiming to obtain protocols or surveillance reports. For this search, no language limitation was applied.

We used Google to identify surveillance programs in countries that were not found through our Medline search and to validate identified programs to obtain publicly available protocols and surveillance reports. We compiled each surveillance program’s characteristics, as not all programs publish their results as peer-reviewed articles.

### Personal communication

When information was not available in official surveillance protocols or on organizations’ websites, an email was sent to authors or program managers to get publicly available documents, such as annual reports of the surveillance performed. A reminder was sent if no answer was received after two weeks from the first communication. Only one reminder was sent.

### Data management

Studies meeting the inclusion criteria were uploaded to DistillerSR (Evidence Partners, Ottawa, Canada), which was used to remove duplicates. Independent screening for title/abstract and full text was performed by the first two authors. If the HAI surveillance program name was available in this section, the information was extracted and validated with a Google search. If the program name was correct, full text review was not performed. If the program name was not found in the title or the abstract for the country, these articles’ full texts were read. Conflicts were resolved through discussion until a consensus was reached.

### Data extraction and quality assessment

An electronic data form was developed on DistillerSR. The following information was extracted from articles, websites and government reports: general information, name of national HAI surveillance programs, HAIs included in the program, jurisdiction, modes of participation (mandatory or voluntary), survey type (incidence or prevalence), reporting periodicity, percentage of facilities involved in the surveillance, microorganisms, medical devices, type of data (individual or aggregated) and official website.

## Results

We identified 6,688 articles with the selected keywords and MeSH. From these, 261 duplicate articles were removed. An additional 6,206 articles were removed because no HAI surveillance program was identified in full-text review. A total of 220 articles (Data S1) were used in this review (Figure S1). Some articles identified programs for more than one country and were counted more than once, which is why the number of articles in Table S2 is 245. The four countries most represented were the US (n=106, 48.2%), Germany (n=31, 14.1%), Spain (n=15, 6.8%) and Italy (n=13, 5.9%).

We identified surveillance programs for 20 of 35 countries. A Google search identified eight additional programs, for a total of 28 of 35 countries (80.0%) having a national program. For the remaining nations (Cyprus, Estonia, Greece, Iceland, Latvia, Malta, and Slovenia), a HAI surveillance program could not be found, but four participated in at least one annual ECDC project. Only 5 of 19 (26.3%) contacted program managers replied (**Table 1** summarizes the information).

**Table 1:** Characteristics of national hospital-acquired infection surveillance programs identified

Program	Country	Type	Frequency of public report	VRE	MRSA	MSSA	CDI	CPE^a^	Gram negative	CLABSI	BSI	SSI	UTI	Venti^b^	ARO	Other
Oceania																
ACSQHC	Australia	I	Annual/quarterly	-	-	-	V	-	-	M,V	M^c^	M,V	-	-	-	-
ANZICS		-	-	-	-	-	-	-	-	M,V	-	-	-	-	-	-
AIHW		-	Annual	-	-	-	-	-	-	-	M^c^	-	-	-	-	-
AGAR		P	Annual	-	-	-	-	-	-	-	-	-	-	-	V^d^	-
North America																
CNISP	Canada	I, P	Annual	V	V	V	V	V	-	V	-	V	-	-	V	V: PPS, *Candida auris*, CSF
NHSN	US	I	Annual	V	V	V	V	V	-	V	-	V	V	V	V	-
United Kingdom																
PHE	England	I	Monthly/annual	-	M^d^	M^d^	M	-	M^d^	-	-	M,V	-	-	-	-
-	North-Ireland	I	Quarterly	-	M	-	M	-	-	-	-	M	-	-	-	V: PPS
WHAIP	Wales	I	Annual/monthly	-	M	-	M	-	-	-	-	M	-	M	-	-
SSHAIP	Scotland	I, P	Quarterly/annual	-	M^d^	-	M	U	M^d^	M^e^	M^e^	M,V	U	M^e^	-	M: norovirus (outbreak), PPS, ICU
Europe																
ANISS	Austria	I	Annual	-	-	-	-	-	-	-	-	V	-	-	-	V: ICU, PPS
NSIH	Belgium	I	Annual	V	M	-	V	-	M	V^e^	M	V	V^e^	M^e^	-	-
-	Croatia	-	-	-	-	-	-	-	-	-	-	-	-	-	-	-
NRC-HAI	Czech Republic	I, P	-	-	-	-	U	-	-	-	-	U	-	-	-	U: PPS, ICU
HAIBA	Denmark	I	Annual	-	-	-	U	-	-	-	U	U	U	-	-	-
DANMAP		P	-	M	M	-	-	M	-	-	-	-	-	-	-	-
HAI-NET	ECDC	I, P	I: Annual P: every five years	-	-	-	V	-	-	-	V^e^	V	V^e^	-	-	V: PPS, V^e^: pneumonia
SIRO	Finland	I	Annual	-	-	-	V	-	-	-	V	V	-	-	-	V: PPS
RAISIN-I	France	I	Annual	-	-	-	-	-	-	-	-	V	-	-	-	-
RAISIN-P		P	Every five years	-	-	-	-	-	-	-	-	-	-	-	-	V: PPS
KISS	Germany	I	Annual	V^f^	V	-	V	-	V^f^	-	V^e^	V	V^e^	V^e^	-	V: neo, LRI^e^
NNSR	Hungary	I	Annual	-	-	-	M	-	-	-	M	V	-	-	M	V: ICU, neonatal M: outbreak
HPSC	Ireland	I	Quarterly/annual	V^d^	V^d^	V^d^	V	M	V^d^	-	-	-	-	V^e^	-	M: PPS
SPIN-UTI	Italy	I	Every two years	-	-	-	-	-	-	V^e^	V^e^	-	V^e^	V^e^	-	-
GiViTi		I	Annual	-	-	-	-	-	-	V^e^	V^e^	-	-	V^e^	-	V^e^: pneumonia
-	Lithuania	I	Annual	-	-	-	-	-	-	-	-	V	-	-	-	V: ICU
-		P	Annual	-	-	-	-	-	-	-	-	-	-	-	-	PPS
NOSIX	Luxembourg	I	-	-	-	-	-	-	-	-	-	-	-	-	-	-
PREZIES	Netherlands	I, P	Annual	-	-	-	-	-	-	M	-	M	-	-	-	V: PPS
SWAB		P	Annual	-	-	-	-	-	-	-	-	-	-	-	V	-
NOIS	Norway	I, P,	Annual	-	-	-	-	-	-	-	M	M	M	-	U	V: PPS, U: LRI, neonatal
-	Poland	I, P	PPS: annual	-	-	-	-	-	-	-	V^e^	-	V^e^	V^e^	-	V: PPS
PPCIRA	Portugal	I	-	-	-	-	-	-	-	V^e^, M^g^	-	V	-	V^e^,M^g^	-	-
EPIS	Slovakia	-	-	-	-	-	-	-	-	-	-	-	-	-	-	-
ENVIN	Spain	I	Annual	-	-	-	-	-	-	V^e^	V^e^	-	V^e^	V^e^	-	-
NEO-KISS		I	-	-	-	-	-	-	-	-	V^g^	-	-	-	-	V^g^: CFS
EPINE		P	Annual	V	V	V	V	V	V	V	V	V	V	V	V	V: PPS
INCLIMECC		I, P	-	-	M	-	M	M	-	M^e^	M^e^	M	M^e^	M^e^	-	M: PPS
SALAR	Sweden	P	Twice a year	-	-	-	-	-	-	-	-	-	-	-	-	M: PPS

Abbreviations: ACSQHC, Australian Commission on Safety and Quality in Health Care; AGAR, Australian Group on Antimicrobial Resistance; AIHW, Australian Institute of Health and Welfare; ANISS, Austrian Nosocomial Infection Surveillance System; ANZICS, Australian And New Zealand Intensive Care Society; ARO, antimicrobial-resistant organisms; BSI, bloodstream infections; CDI, *Clostridoides difficile*; CLABSI, central line-associated bloodstream infection; CNISP, Canadian Nosocomial Infection Surveillance Program; CPE, carbapenemase-producing Enterobacteriaeceae; CPO, carbapenemase-producing organisms; CRE, carbapenem-resistant Enterobacteriaceae; CSF, cerebrospinal fluid shunt; DANMAP, Danish Integrated Antimicrobial Resistance Monitoring and Research Programme; ECDC, European Centre for Disease Prevention and Control; ENVIN, Estudio Nacional de Vigilancia de Infección Nosocomial en Servicios de Medicina Intensiva; EPINE, study on the prevalence of nosocomial infections in Spain; EPIS, national epidemiologic surveillance systems; GiViTi, Gruppo italiano per la Valuatazion degli interventi In Terapia intensiva; HAIBA, Healthcare-Associated Infections Database; HAI-NET, Healthcare-Associated Infections Surveillance Network; HPSC, Health Protection Surveillance Center; I, incidence; ICU, intensive care unit; INCLIMECC, Indicadores Clínicos de Mejora Continua de la Calidad; KISS, German Nosocomial Infection Surveillance System; LRI, lower respiratory infection; M, mandatory; MRSA, methicillin-resistant *Staphylococcus aureus* bloodstream infections; MSSA, methicillin-susceptible *Staphylococcus aureus* bloodstream infection; NEO-KISS, Neonatology-KISS; NHSN, National Healthcare Safety Network; NNSR, National Nosocomial Surveillance System; NOIS, surveillance system for hospital acquired infections; NOSIX, Luxembourg Nosocomial Infection Surveillance System; NRC-HAI, National Reference Center for Healthcare Associated Infections; NSIH, National Surveillance of Healthcare associated and antimicrobial resistance; P, prevalence; PHE, Public Health England; PPCIRA, Programa de Prevenção e Controlo de Infeções e de Resistência aos Antimicrobianos; PPS, point prevalence survey; PREZIES, Prevention of Nosocomial Infection through Surveillance; RAISIN, Réseau d’alerte, d’investigation et de surveillance des infections nosocomiales; SALAR, Swedish Association of Local Authorities and Regions; SIRO, Finnish Hospital Infection Programme; SPIN-UTI, Italian Nosocomial Infections Surveillance in Intensive Care Units; SSHAIP, Scottish Surveillance of Healthcare Associated Infection Programme; SSI, surgical site infection; SWAB, Dutch Working Party on Antibiotic Policy; U, unknown; US, United States; UTI, urinary tract infection; V, voluntary; VAE, ventilator-acquired event; VAP, ventilator-acquired pneumonia; VRE, vancomycin-resistant enterococci; WHAIP, Welsh Healthcare Associated Infection Programme; -, not applicable

^a^ CPE and/or CPO and/or CRE

^b^ VAP and/or VAE

^c^ *Staphylococcus aureus*

^d^ Sepsis

^e^ ICU

^f^ ARO

^g^ Neonatal

We kept information from national HAI surveillance programs as we aimed to understand how alliance of regions pooled data; thus, programs that were not at least national in scope and those for which we could not differentiate between community-acquired or hospital-acquired infections were excluded.

### Surveillance programs

We identified 38 national HAI surveillance programs for 28 countries with a national surveillance program (Table 1). Some countries have two or more surveillance programs. Most national surveillance programs reported yearly incidence on a voluntary basis. Surgical site infection (SSI) surveillance was done in 21 of 35 countries (**Table 2**). Infections and procedures under surveillance are detailed in Table 1. Twenty-six programs for which data was available used active surveillance. None reported use of administrative surveillance as the only source of data.

**Table 2:** National surveillance programs for surgical site infections, 21 countries

Program	Country	CABG	Laparoscopic CHOL	Open CHOL	Laparoscopic COLO	Open COLO	CSEC	HPRO	KPRO	LAM	Other
ACSQHC	Australia	X	-	-	-	-	X	X	X	X	Appendectomy, cholecystectomy, colectomy, craniotomy, hernia repair and spinal fusion
CNISP	Canada	-	-	-	-	-	-	X	X	-	Paediatric cardiac surgery
NHSN	US	X	X	X	X	X	X	X	X	X	30 more
NINSS	England	X	-	-	-	-	-	X	X	-	Abdominal hysterectomy, bile duct, liver or pancreatic, breast, cardiac surgery (non-CABG), cholecystectomy, cranial, gastric, large bowel, limb amputation, reduction of long bone fracture, repair of neck of femur, small bowel, spinal, vascular
-	North Ireland	-	-	-	-	-	X	X	X	X	-
WHAIP	Wales	-	-	-	-	-	X	X	X	-	-
SSHAIP	Scotland	X	-	-	-	-	X	X	X	-	Abdominal hysterectomy, breast, cardiac, cranial, large bowel, reduction of long bone fracture, repair of neck of femur, vascular
ANISS	Austria	X	X	X	X	X	X	X	X	-	Abdominal hysterectomy, appendectomy, ear nose throat, genitourinary, herniorrhaphy, kidney, mastectomy, prostate, skin (correctional and scar), small bowel and vaginal hysterectomy
NSIH	Belgium	X	X	X	X	X	X	X	X	X	-
NRC-HAI	Czech Republic	-	-	-	-	-	-	-	-	-	Site under construction
HAIBA	Denmark	-	-	-	-	-	-	X	X	-	-
HAI-NET	ECDC	X	X	X	X	X	X	X	X	X	-
SIRO	Finland	-	-	-	-	-	-	X	X	-	Paediatric open heart
RAISIN	France	X	X	X	X	X	X	X	X	X	Bariatric, coronary, orthopedic, digestive, neurosurgery, obstetric gynecology, reconstructive, thoracic, traumatological, urological and vascular
KISS	Germany	X	X	X	X	X	X	X	X	X	-
NNSR	Hungary	X	X	X	X	X	X	X	X	X	Abdominal hysterectomy, appendectomy, cardiac, limb amputation, reduction of long bone fracture
-	Lithuania	X	X	X	X	X	X	X	X	-	Appendix, inguinal hernia, orthopedic, traumatological, vascular (venous)
PREZIES	Netherlands	X	X	X	X	X	X	X	X	X	Breast, femoral head replacement, isolated open aortic valve, pacemaker implantation
NOIS	Norway	X	X	X	X	X	X	X	-	-	-
PPICRA	Portugal	X	X	X	X	X	X	X	X	X	-
EPINE	Spain	X	X	X	X	X	X	X	X	X	30 more
INCLIMECC		X	-	-	X	X	-	X	X	-	Appendectomy, fusion vertebral, gastric, herniorrhaphy, rectum
Total: N (%)	-	15 (71.4%)	12 (57.1%)	12 (57.1%)	12 (57.1%)	12 (57.1%)	16 (76.1%)	20 (95.2%)	19 (90.5%)	11 (52.4%)	-

Abbreviations: ACSQHC, Australian Commission on Safety and Quality in Health Care; ANISS, Austrian Nosocomial Infection Surveillance System; CABG, coronary artery bypass graft; CHOL, cholecystectomy; CNISP, Canadian Nosocomial Infection Surveillance Program; COLO, colon surgery; CSEC, caesarean section; ECDC, European Centre for Disease Prevention and Control; EPINE, study on the prevalence of nosocomial infections in Spain; HAIBA, Healthcare-Associated Infections Database; HAI-NET, Healthcare-Associated Infections Surveillance Network; HPRO, hip prosthesis surgery; INCLIMECC, Indicadores Clínicos de Mejora Continua de la Calidad; KISS, German Nosocomial Infection Surveillance System; KPRO, knee prosthesis surgery; LAM, laminectomy; NHSN, National Healthcare Safety Network; NINSS, Nosocomial Infection National Surveillance Scheme; NNSR, National Nosocomial Surveillance System; NOIS, surveillance system for hospital acquired infections; NRC-HAI, National Reference Center for Healthcare Associated Infections; NSIH, National Surveillance of Healthcare associated and antimicrobial resistance; PPCIRA, Programa de Prevenção e Controlo de Infeções e de Resistência aos Antimicrobianos; PREZIES, Prevention of Nosocomial Infection through Surveillance; RAISIN, Réseau d’alerte, d’investigation et de surveillance des infections nosocomiales; SIRO, Finnish Hospital Infection Programme; SSHAIP, Scottish Surveillance of Healthcare Associated Infection Programme; US, United States; WHAIP, Welsh Healthcare Associated Infection Programme;X, surveillance done by the country; -, not applicable

The HAI surveillance network (HAI-NET) from the ECDC performs two types of surveillance: 1) a point prevalence survey (PPS) of HAIs in European acute care hospitals ((7)), every five years; and 2) three annual incidence surveillance for *Clostridoides difficile* infections (CDI) ((8)), infections acquired in intensive care unit (ICU) ((9)) and SSI ((10)) (Table S3). In total, 33 countries/regions (29 ECDC countries and four UK regions) participated in the PPS ((7,11,12)). Four periods were selected for data collection (April–June and September–November of each year), avoiding the summer holidays (lower staffing) and the winter period (higher antimicrobial use). Denominator data could be either: patient-based (optional) or unit-based (mandatory). Patient present on the ward at 8 a.m. and not discharged during the survey were counted in the denominator.

The SSI surveillance included nine surgical procedures: coronary artery bypass graft; open and laparoscopic cholecystectomy; open and laparoscopic colon surgery; caesarean section; hip prosthesis; knee prosthesis; and laminectomy, according to three case definitions: superficial incisional; deep incisional; and organ/space ((13)). Two indicators are produced: 1) proportion of SSIs by surgical procedure category (HAI/specific surgical procedure) in the 30 days after surgery, if no implant, and in the 90 days, if implant; and 2) proportion of SSIs diagnosed before hospital discharge. Fifteen countries/regions participated to the last annual surveillance ((10)).

The CDI surveillance was recommended as a continuous surveillance over 12 months, with a minimal duration of three consecutive months ((14)). The denominator includes all hospitalized patients regardless of age. Every case meeting the case definition is included in the numerator. According to the last available published report, 20 countries/regions participated in the surveillance ((8)).

In the last ECDC ICU-based surveillance, 11 countries/regions participated ((9)). Five infections were included: pneumonia, bloodstream infection (BSI), urinary tract infection (UTI), device-related infections (e.g. ventilator-associated pneumonia [VAP], central line-associated bloodstream infection [CLABSI], catheter-associated CA-UTI), catheter-related infection (CRI), and other HAI (including neonatal infections). Two surveillance options were available: unit-based and patient-based ((15)). To be considered in the denominator, a patient must stay for at least three days in the ICU. HAI surveillance is recommended for three to six months each year.

The National Healthcare Safety Network (NHSN, US) is separated in six components with HAI surveillance included in Patient Safety ((16)). Participating hospitals must produce a monthly reporting plan of what will be under surveillance and an annual facility survey. In acute care, six infections/procedures are monitored: CLABSI, CA-UTI, ventilator associated event (VAE) and paediatric VAE, SSI, multidrug-resistant organisms (MDRO) and CDI ((16)).

Many rates are produced for CDI and MDRO ((16)). For MDRO, prevalence rates are calculated for inpatients, community onset, healthcare facility onset, and outpatients, MDRO infection/colonization incidence or incidence density rates are also calculated (**Table 3**). In the last NHSN report ((17)), all states/territories reported at least one acute care facility for one month of data for every infection. The state’s mandate for NHSN varies by infection (**Table 4**).

**Table 3:** Numerator and denominator information collected per hospital-acquired infections for rate calculations, National Healthcare Safety Network, U.S., 2021

HAI	Numerator	Denominator	Outcomes		
			Incidences rates		Device utilization ratio
CLABSI	# infections	Device-days	# infections/# central-line days	X 1,000	# central-line days/# patient days
		Patient-days			
Pneumoniae	# infections	Device-days	# VAP/# ventilation-days	X 1,000	# ventilation-days/# patient days
		Patient-days			
CA-UTI	# infections	Device-days	# infections/# catheter-days	X 1,000	# catheter-days/# patient days
		Patient-days			
SSI	# infections: superficial, deep, organ/space	All patients for each procedure	# SSI/# specific procedure	X 100	-
VAE or PedVAE	# infections	Device-days	# VAE/# ventilation-days	X 1,000	# ventilation-days/# patient days
		Patient-days			
MDRO	Laboratory confirmed MDRO Healthcare facility onset	Admission	# MDRO BSI/# admission	X 100	-
		Patient-days	# MDRO BSI/# patient-days	X 1,000	
CDI	Laboratory confirmed CDI	Patient-days	# CDI/# patient-days	X 10,000	-
	Community-onset healthcare facility associated		# CDI HO/# patient-days		
	Healthcare facility onset		# CDI (HO + CO-HCFA)/# patient-days		

Abbreviations: BSI, bloodstream infection; CA-UTI, catheter-associated urinary tract infection; CDI, *Clostridoides difficile* infection; CLABSI, central line-associated bloodstream infection; CO-HCFA, community-onset healthcare facility associated; HAI, healthcare-associated infections; HO, healthcare facility onset; MDRO, multidrug-resistant organisms; PedVAE, paediatric ventilator-associated event; SSI, surgical site infection; VAE, ventilator-associated event; VAP, ventilator-associated pneumonia; -, not applicable

**Table 4:** States with mandatory participation in US National Healthcare Safety Network, surveillance program, per infection, 2018

Mandated	CLABSI	CA-UTI	VAE	COLO	HYST	MRSA	CDI
Yes	29	23	6	25	24	23	25
No	15	21	38	19	20	21	19
Unknown	10	10	10	10	10	10	10

Abbreviations: CA-UTI, catheter-associated urinary tract infections; CDI, *Clostridoides difficile* infection; CLABSI, central line-associated bloodstream infection; COLO, colon surgery; HYST, hysterectomy; MRSA, methicillin-resistant *Staphylococcus aureus* bloodstream infections, VAE, ventilator-associated event

To compare each state’s performance, NHSN calculated the specific standardized infection ratio (SIR) each year ((17)), which is “the ratio of the observed number of infections to the number of predicted infections per year” ((18)). Three benchmarks are compared with each state’s annual SIR: the current national SIR (removing specific state from national SIR), the state’s SIR from 2019, and the 2015 national baseline ((17)).

The Canadian Nosocomial Infection Surveillance Program (CNISP) included 87 of 620 Canadian hospitals (14.0%) in 2021 ((19)) and performs two types of surveillance: 1) PPS of HAIs with an estimation of the proportion of infections caused by AROs ((4)), which is done approximately every seven years; and 2) annual incidence surveillance for HAIs.

CNISP conducted three PPS (2002, 2009 and 2017). In the last report, 47 of 66 (71.2%) invited hospitals participated ((4)). Data collection included hospital profile, patients’ demographic data and information on HAI. In 2017, data were collected for VAP, SSI (hip and knee), UTI, methicilin-resistance *Staphylococcus aureus* (MRSA), vancomycin-resistant enterococci (VRE), extended-spectrum beta-lactamase producing organisms, carbapenemase-producing organisms (CPO) and CDI. Surveillance includes patients of any age admitted to the hospital for at least 48 hours, or for less than 48 hours if they were admitted in the month prior to the survey.

Several infections were part of the annual incident surveillance: *Candida auris*; CDI; CLABSI; CPO; SSI (knee, hip, cardiac [paediatric], and cerebrospinal fluid shunt); and methicillin-susceptible *Staphylococcus aureus* (MSSA), MRSA and VRE BSI. Patient-level data were collected for all infections, except for CDI for which aggregated data could be submitted (minimum dataset). Hospitals chose which surveillance program they participated in. For example, in 2018, 62 hospitals participated in the MRSA and VRE BSI surveillance, 59 to carbapenemase-producing Enterobacteriaeceae (CPE) and 68 to CDI ((20)).

In Australia, the Australian Commission on Safety and Quality in Health Care (ACSQHC) was established in 2011. It develops protocols used by other groups, such as the Australian Institute of Health and Welfare and the Australian and New Zealand Intensive Care Society, to perform surveillance. Established in 1985, the Australian Group on Antimicrobial Resistance performs the surveillance for antimicrobial resistance (MSSA, MRSA, VRE, gram-negative bacteria and CPE) in blood. This voluntary surveillance is based on laboratories’ participation.

Public Health England does surveillance for CDI, bacteremia (gram-negative, MRSA and MSSA) and SSI. The SSI surveillance is mandatory for orthopedic surgery for a minimum of three consecutive months per fiscal year ((21)). The other surveyed procedures are voluntary (Table 2). Patients are followed for 30 days (non-implant procedures) and one year (prosthetic implant procedures). In the last available report, 156 hospitals reported for hip and knee replacements. In comparison, only 20 and 16 hospitals reported for large bowel surgery and spinal and breast surgeries (voluntary program), respectively. Public Health England analyzes submitted data quarterly to identify high (hospitals whose SSI risk is greater than the 90^th^ percentile) and low outliers (less than the 10^th^ percentile). Low outliers are supported to ensure all cases are being reported. High outliers are asked to explore their clinical practices to identify possible reasons to explain high rates. CDI and BSI are mandatory programs ((22)). Public Health England receives data from all hospitals and publicly shares monthly or annual rates on their website.

## Discussion

The objective of this scoping review was to synthesize characteristics of national HAI surveillance programs from 35 selected countries to inform decisions on possible national programs for Canada. Most surveillance was done on a voluntary basis. CDI, hip and knee prosthesis surgery and caesarean sections were the four main infections and procedures under surveillance.

Characteristics of surveillance programs appear to vary, including for frequency of reporting to ministries. Some countries use prevalence point surveys as their main method of surveillance. The percentages of participating hospitals vary (from 1.4% to 100%). With 9.5% to 11.0% participation, CNISP is in the lower range (Table S4). Double data entry (at the hospital and at the national program level) can be a barrier to participation, given the additional workload. From the 18 programs with available information, 16 (88.9%) required double data entry through forms to collect data. Finally, all surveillance program with available information used active surveillance and 77.8% reported data at the hospital level (n=21/27, data not shown).

The published reports reviewed for this scan did not describe how benchmarks were set; for example, in Lithuania, a national average of infection rates was used as a threshold for comparison. In Australia since 2016–2017, the National Healthcare Agreement sets a national benchmark of less than or equal to 1.0 HAI of *S. aureus* BSI per 10,000 bed days ((23,24)). The NHSN goes further by stratifying benchmarks according to patient population; for example, an NSHN surveillance report in 2006–2007 separated average rates by different characteristics ((25)). Data collected in ICUs, specialty care area or wards were stratified by patient population: adult or paediatric. Data collected on infections from neonatal ICU are stratified by birthweight categories. The VAP and CLABSI rates are stratified by department or ICU type (e.g. trauma, surgical). Greater precision in benchmarks allows for a better understanding of where interventions are needed, by allowing for more refined comparisons.

In view of other national surveillance programs, some elements must be considered for HAI surveillance in Canada. Although provinces have their own surveillance programs, there is a need for large enough sample sizes to stratify infection rates for specific units (e.g. cardiac, neonatal or paediatric ICUs): this will require data to be pooled at the national level. Data transmitted from provinces to the federal surveillance program could be aggregated, but numerators and denominators and harmonized surveillance definitions are required. The CNISP is currently using harmonized definitions across the country with patient or unit-level data, but it currently lacks representativeness, as it represents only a fraction of Canadian healthcare, with a bias towards teaching urban hospitals. Recruitment of new hospitals into CNISP requires funding. Voluntary participation of all Canadian hospitals in CNISP is being considered but the risk of selection bias remains.

### Limitations

This study has several limitations. First, for us to be able to identify a program, the country must publicly report it. Non-English websites or grey literature reports were translated using two tools. The first was the internet navigator itself, using Google Chrome tools for website translation. The second tool was the software DeepL Translator (DeepL, Cologne, Germany). Although these tools may have inherent limitations, data extracted were objective and straightforward and did not require any subtle interpretation. The risk of selection bias from published literature was mitigated by a web search for each identified country. Although we may have missed some smaller national programs, we think that most elements of a HAI surveillance program have been captured via larger national or multinational programs, such as ECDC. Other information (process and not results) was extracted from official available protocols and reports from the program website or by speaking to the program’s manager.

## Conclusion

In the four regions studied, 80% of high-income countries had national HAI surveillance programs. Although some differences exist, the overarching theme was that national surveillance programs had individual-level data, or at least aggregated data at a hospital level, with a numerator and a denominator and not just an overall incidence rate by region. Infections and procedures under surveillance are quite uniform. This literature scan is the first step towards identifying the best approach for a national HAI surveillance program for Canada.

## Supplemental material

These documents can be accessed on the Supplemental material file.
Table S1: Keywords and MeSH classification of the four concepts identified for the scoping review
Data S1: List of 220 articles identified by systematic review through Medline
Figure S1: Flow chart of the scoping review
Table S2: Number of articles found by countries
Table S3: Number of participating hospitals for four HAI-NET’s surveillance programs, European Centre for Disease Prevention and Control
Table S4: Infections surveyed in national surveillance programs, 37 countries, 2021
